# Stair-climbing resistance exercise, with or without oral turmeric (*Curcuma longa* L.) supplementation, relieves symptoms and promotes musculoskeletal repair in experimental rheumatoid arthritis in rats

**DOI:** 10.1007/s10974-026-09726-1

**Published:** 2026-03-13

**Authors:** Mikael Gerson Kuhn, Leticia Massochim da Silva, Flávia Heloísa da Silva, Maiara Cristina Lima de Jesus, Michele Gossler, Gabriella Lopes Cappellaro, Gladson Ricardo Flor Bertolini, Lucinéia de Fátima Chasko Ribeiro, Ana Tereza Bittencourt Guimarães, Marcia Miranda Torrejais

**Affiliations:** 1 Programa de Pós-Graduação em Biociências e Saúde – CCBS, Universidade Estadual do Oeste do Paraná (Unioeste), Rua Universitária, PR 85819-110 Cascavel, Brasil; 2 Faculdades Pequeno Príncipe, Av. Iguaçu 333, Curitiba, PR 80230-020 Brasil

**Keywords:** Adjuvant-induced arthritis, Resistance exercise, Turmeric, Skeletal muscle, Neuromuscular junction

## Abstract

Rheumatoid arthritis is a chronic systemic inflammatory disease associated with joint inflammation, skeletal muscle dysfunction, and reduced physical capacity, reinforcing the need for complementary non-pharmacological strategies aimed at preserving musculoskeletal function. This study investigated the functional and morphological effects of turmeric supplementation associated with stair-climbing resistance exercise in *Wistar* rats with adjuvant-induced arthritis (AIA), an experimental model representative of rheumatoid arthritis. Thirty-one *Wistar* rats were randomly allocated into five groups: control (CTRL, *n* = 6), adjuvant-induced arthritis (AIA, *n* = 6), AIA treated with turmeric (AIA + T, *n* = 6), AIA subjected to stair-climbing resistance exercise (AIA + E, *n* = 6), and AIA treated with turmeric combined with exercise (AIA + T+E, *n* = 7). Arthritis was induced using Complete Freund’s Adjuvant. Functional outcomes included joint swelling and hindlimb grip strength. On Day 23, animals were euthanized, and analyses of muscle fibers, connective tissue, and neuromuscular junctions were performed. AIA induced joint swelling and reduced muscle strength. Histological analyses revealed muscle fiber alterations characterized by fiber size variability, centrally located nuclei, and mononuclear inflammatory infiltration. These alterations were less pronounced in exercise-treated groups. Stair-climbing resistance exercise improved functional performance and preserved muscle morphology, whereas turmeric supplementation alone produced limited functional effects. Neuromuscular junction morphometry showed an increase in smaller diameter in arthritic animals that was attenuated by exercise. Stair-climbing resistance exercise attenuated functional and morphological impairments associated with adjuvant-induced arthritis, supporting its role as a complementary therapeutic strategy. Turmeric supplementation alone showed limited effects under the conditions tested and warrants further investigation.

## Introduction

Rheumatoid arthritis (RA) is a chronic, progressive, and symmetrical autoimmune disease that primarily affects small joints and may extend to larger joints, also compromising surrounding tissues such as skeletal muscle (Andrade et al., 2019). It affects approximately 1% of the global population, and its incidence has increased with population ageing (Andrade et al., 2019). Clinical manifestations include joint swelling, warmth, and persistent pain during disease flares. The inflammatory process is characterized by cytokine accumulation and immune cell infiltration, leading to synovitis and contributing to muscle catabolism and sarcopenia through mediators such as TNF-α (tumor necrosis factor alpha) and IL-6 (interleukin 6) (Moschou et al. 2023).

Although pharmacological therapies effectively control disease activity, they may be associated with adverse effects and high costs, supporting the search for complementary strategies aimed at preserving musculoskeletal function and improving quality of life (Passos 2016). Among these, turmeric (*Curcuma longa* L.) has gained attention due to its anti-inflammatory and antioxidant properties, with evidence suggesting potential benefits in reducing pain and inflammatory markers in RA (Hewlings and Kalman, 2017).

Physical exercise also plays a relevant role in RA management. Resistance training improves muscle strength, hypertrophy, and power, and when properly prescribed, promotes adaptive responses that may mitigate sarcopenia and enhance joint function. Additionally, it contributes to cartilage preservation, mobility maintenance, and neuromuscular activation (Mackey et and Kjaer, 2017).

However, few studies have investigated the combined effects of turmeric supplementation and stair-climbing resistance exercise on skeletal muscle under inflammatory conditions. Therefore, the aim of this study was to evaluate the functional and morphological effects of turmeric supplementation associated with stair-climbing resistance exercise in *Wistar* rats with adjuvant-induced arthritis (AIA), an experimental model representative of rheumatoid arthritis.

## Methods

This experimental, quantitative, randomized study was approved by the Ethics Committee for the Use of Animals of the State University of Western Paraná (protocol no. 13–22). All procedures were conducted at UNIOESTE (Cascavel campus) at the Laboratory for the Study of Injuries and Physiotherapeutic Resources and the Experimental Morphology Laboratory, in accordance with the ARRIVE guidelines 2.0 (Percie du Sert et al. 2020).

### Obtaining animals and experimental design

Thirty-one male *Wistar* rats (3 months old at the beginning of the experimental protocol; mean body weight 340 g) were obtained from the UNIOESTE Central Animal Facility and acclimatized for 1 week. Animals were housed according to treatment groups, with turmeric-treated groups kept separately to avoid cross-contamination. Rats were randomly allocated into five groups: control (CTRL; *n* = 6), adjuvant-induced arthritis (AIA; *n* = 6), AIA + turmeric (AIA + T; *n* = 6), AIA + stair-climbing resistance exercise (AIA + E; *n* = 6), and AIA + turmeric + exercise (AIA + T+E; *n* = 7).

Sample size was estimated based on an ANOVA model (power 90%, α = 0.01) using G*Power 3.1.9.7 (Windows).

### Adjuvant-induced arthritis

Complete Freund’s Adjuvant (CFA) (Gomes et al. 2014) was used to induce adjuvant-induced arthritis. Rats received 50 µL of CFA (0.5 mg/mL; Mycobacterium butyricum; Difco^®^) intradermally at the base of the tail. Control animals received saline (0.9% NaCl; Aster^®^). Seven days later, an intra-articular injection (50 µL CFA or saline for controls) was performed in the tibiofemoral joint of the right hindlimb under aseptic conditions using 1% iodized alcohol (Rialcool^®^).

For both injections, animals were gently restrained using a flannel cloth, as approved by the institutional Ethics Committee for the Use of Animals (protocol no. 13–22), and no anesthesia was used.

Animals were included in the arthritic groups only if visible tibiofemoral joint swelling was present 24 h after the intra-articular injection. Animals without joint swelling were excluded (*n* = 0).

### Turmeric supplementation protocol

Turmeric supplementation was administered to AIA + T and AIA + T+E groups starting 24 h after intra-articular injection and continued for 15 days (Nonose et al. 2014). Animals received 100 mg/kg/day of *Curcuma longa* L. dry extract by gavage, with doses adjusted weekly according to body weight. CTRL, AIA, and AIA + E received water by gavage to control for handling stress.

### Stair-climbing resistance exercise protocol

Exercise adaptation was performed before arthritis induction, consisting of **7** consecutive days of climbing (nine climbs per session, without load) to familiarize animals with the ladder apparatus (Jacob et al. 2022). The adaptation protocol was completed prior to the first CFA injection. The apparatus was a vertical wooden ladder (1.18 m high, 20.5 cm wide, 70° incline) with 67 iron rungs and a rest box at the top.

After adjuvant-induced arthritis was established, the resistance training period was initiated 24 h after the intra-articular injection and continued for 15 days, performed every other day. Each session consisted of three sets of eight climbs with progressive loads (25%, 35%, and 50% of body weight) using lead plates attached to the tail, with 2-min rest intervals between sets.

### Functional assessments

All groups underwent joint swelling and grip strength assessments. During the adaptation week, animals were familiarized with the assessment equipment for 5 days. Measurements were performed blinded by the same researcher at the same time of day at five time points: baseline (BA; Day 1), after induction (FA.1; Day 11), and during follow-up (FA.2–FA.4; Days 15, 19, and 23).

#### Assessment of joint swelling

To assess joint swelling, the animals were restrained with a flannel for the duration of the measurement. Joint swelling was assessed by measuring the femoral–tibial joint diameter of the right hindlimb along the medial–lateral axis using a caliper (Mister^®^, Rio Grande do Sul, Brazil), according to Neves et al. (2020). Three measurements were obtained per animal, and the mean value was used (cm).

#### Grip strength assessment (muscle strength)

Grip strength was assessed in the right hindlimb using a grip strength meter (Insight^®^, Ribeirão Preto, São Paulo), according to Coradinia et al. (2015). The animal was gently pulled by the tail and trunk, allowing it to grasp the grid connected to the force transducer with the right hindlimb until it lost its grip. The peak force was recorded (g).

### Euthanasia of the animals

On Day 23, at the end of the experimental period, the rats (4 months old) were weighed, measured (nasoanal length), and had the Lee index calculated. Euthanasia was performed via intraperitoneal injection of ketamine hydrochloride (240 mg/kg, Ketalar^®^, Brazil) and xylazine (45 mg/kg, Xilazin^®^, Brazil).

### EDL muscle collection

The right extensor digitorum longus (EDL) muscle was collected for morphological and morphometric analyses. After removal of the tibialis anterior, the EDL was dissected, measured with a digital caliper (Digimess^®^, Brazil), and weighed on an analytical scale (Bel^®^, Brazil). Samples were divided for histological and histochemical analyses.

### Histological studies of muscle fibers and connective tissue

The proximal segment of the right EDL was fixed in Metacarn for 24 h and stored in 70% ethanol. Samples were dehydrated in graded alcohol series, cleared in n-butyl alcohol, embedded in paraffin, and cross-sectioned (7 μm) using a Leipzig microtome.

Sections were stained with hematoxylin and eosin (Junqueira and Junqueira 1983). Muscle fiber nuclei, fiber area, largest and smallest diameters, and the capillary-to-fiber ratio were assessed in 10 fields per animal using a 40× objective. Capillaries were counted by two blinded assessors following Fernandes et al. (2012).

A histopathological index was applied (Zazula et al. 2022), considering lesion importance (w = 1–3) and extent (α = 0–6), with total score calculated as x = α*w (maximum = 320).

Connective tissue was evaluated using Masson’s trichrome staining (Bancroft and Stevens 1990). Ten microscopic fields per animal were analyzed using a 40× objective to quantify connective tissue as a percentage of total tissue area. For neuromuscular spindle analysis, all observable spindles in a section were counted under a 40× objective.

### Morphological and morphometric study of the neuromuscular junctions

For neuromuscular junction evaluation, the distal segment of the right EDL was immersed in Karnovsky fixative (Karnovsky 1964) and stored refrigerated until processing. The muscle was longitudinally sectioned into 3–4 slices using stainless steel blades and subjected to the nonspecific esterase reaction (Lehrer and Ornstein 1959). Because this histochemical protocol requires free-hand thick sections, image acquisition has inherent limitations regarding focal depth. All images were acquired under identical optical conditions.

Neuromuscular morphometry (area, largest diameter, and smallest diameter) was obtained from 100 NMJs per animal from images acquired using a 20× objective.

### Image acquisition and analysis

The morphological and morphometric analyses of the EDL muscle were based on images obtained by an Olympus DP71 camera (Tokyo, Japan) coupled to an Olympus Bx60^®^ microscope with the aid of the DP Controller 3.2.1 276 program. The images were analyzed using Image Pro Plus 6.0^®^ (Media Cybernetics, Maryland, USA).

### Statistical analysis

The animal was considered the experimental unit (biological replicate), with 6–7 animals per group. For morphometric and histological analyses, multiple fields/structures were evaluated per animal and averaged to yield one value per animal for statistical comparisons.

Parametric body, morphometric, and histological data are presented as mean ± SD and were analyzed using Student’s t-test when comparing CTRL and AIA (to confirm arthritis induction).

Comparisons among arthritic groups (AIA, AIA + T, AIA + E, and AIA + T+E) were performed using two-way ANOVA followed by Fisher’s post hoc test, when appropriate.

Non-parametric data were analyzed using the Kruskal–Wallis test followed by Dunn’s post hoc test.

Functional outcomes (joint swelling and grip strength) were analyzed using two-way repeated-measures ANOVA (time as within-subject factor and group as between-subject factor) and are presented as mean ± SEM, followed by Fisher’s post hoc test. Statistical significance was set at *P* < 0.05. Analyses were performed using XLStat 2014 (Addinsoft^®^, Paris, France).

## Results

### Body parameters

No significant differences were observed in body weight, nasoanal length, EDL muscle weight, or EDL muscle length when comparing the AIA group with CTRL. However, the Lee index was reduced by 2% in AIA compared with CTRL (*P* = 0.0199). No significant differences were observed among the remaining experimental groups (Table 1).

**Table 1:** Body parameters of 4-month-old *Wistar* rats

Body parameters	CTRL (*n* = 6)	AIA (*n* = 6)	AIA + T (*n* = 6)	AIA + E (*n* = 6)	AIA + T+E (*n* = 7)
Body weight (g)^1,3^	326 ± 54	311 ± 25	323 ± 25	334 ± 25	313 ± 27
Nasoanal length (cm)^1,3^	23.1 ± 1.5	23.3 ± 0.5	23.1 ± 1.2	23.5 ± 0.7	23.2 ± 0.6
Lee Index^1,3^	297 ± 5ᵃ	290 ± 2ᵇ	290 ± 13ᵇ	294 ± 4ᵇ	290 ± 8ᵇ
EDL muscle weight (g)^1,3^	0.14 ± 0.02	0.14 ± 0.03	0.12 ± 0.02	0.14 ± 0.04	0.13 ± 0.04
EDL muscle length (cm)^1,3^	2.0 ± 0.4	1.9 ± 0.4	1.8 ± 0.4	1.8 ± 0.3	1.9 ± 0.7

CTRL control group; AIA adjuvant-induced arthritis; AIA + T adjuvant-induced arthritis + turmeric; AIA + E adjuvant-induced arthritis + stair-climbing resistance exercise; AIA + T+E adjuvant-induced arthritis + turmeric + stair-climbing resistance exercise; EDL extensor digitorum longus

Values are expressed as mean ± standard deviation. Different superscript letters indicate statistically significant differences (*P* < 0.05)

¹ Student’s t-test (parametric data; AIA vs. CTRL)

² Mann–Whitney test (non-parametric data; AIA vs. CTRL)

³ Two-way ANOVA with Fisher’s post hoc test (parametric data among AIA, AIA + T, AIA + E, and AIA + T+E)

⁴ Kruskal–Wallis test with Dunn’s post hoc test (non-parametric data among AIA, AIA + T, AIA + E, and AIA + T+E)

### Functional assessments

#### Assessment of joint swelling

Significant effects were observed for time (F(4,130) = 467.37, *P* < 0.0001), group (F(4,130) = 12.05, *P* < 0.0001), and the time **×** group interaction (F(16,130) = 12.29, *P* < 0.0001).

**Fig. 1:**
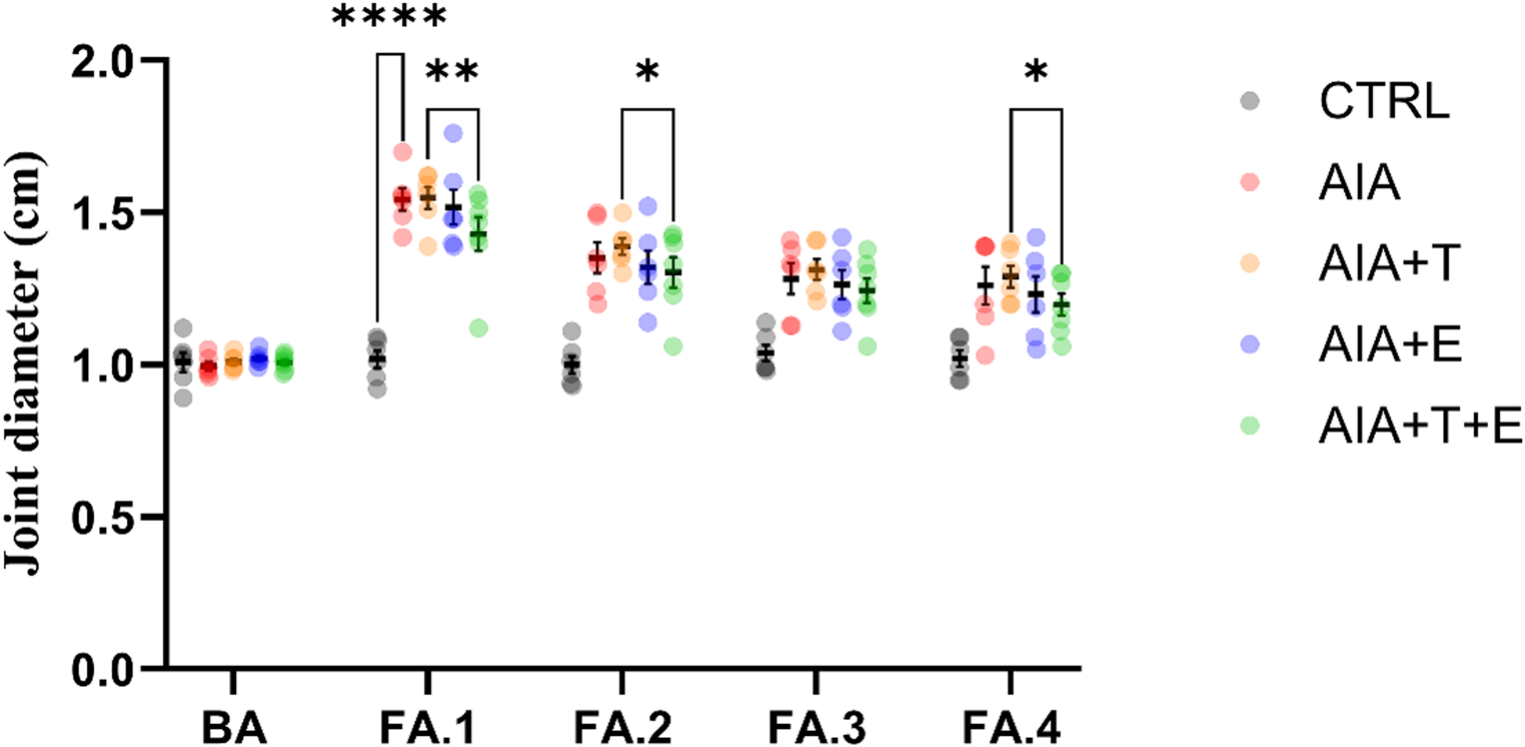
Femoral–tibial joint diameter (cm) in 4-month-old *Wistar* rats. Experimental groups: control (CTRL, *n* = 6), adjuvant-induced arthritis (AIA, *n* = 6), AIA + turmeric (AIA + T, *n* = 6), AIA + stair-climbing resistance exercise (AIA + E, *n* = 6), and AIA + turmeric + exercise (AIA + T+E, *n* = 7). Measurements were performed at baseline (BA; Day 1), after induction (FA.1; Day 11), and during follow-up (FA.2–FA.4; Days 15, 19, and 23). Each dot represents one animal (biological replicate). Horizontal bars indicate the mean and vertical bars indicate mean ± SEM. Data were analyzed using two-way repeated-measures ANOVA followed by Fisher’s post hoc test. For clarity, brackets indicate selected key between-group comparisons at the indicated time points (CTRL vs. AIA at FA.1; AIA + T+E vs. AIA + T at FA.1, FA.2, and FA.4); all other comparisons are reported in the Results text. *P < 0.05, **P < 0.01, ***P < 0.001, ****P < 0.0001

Across follow-up time points (FA.1–FA.4), the AIA group exhibited greater femoral–tibial joint swelling than CTRL (*P* < 0.0001). AIA + T and AIA + E did not differ from AIA. In contrast, AIA + T+E showed reduced swelling compared with AIA + T at FA.1 (*P* = 0.0025), FA.2 (*P* = 0.0302), and FA.4 (*P* = 0.0168), and compared with AIA + E at FA.1 (*P* = 0.0220) (Fig. 1).

Within-group analyses revealed no changes in CTRL. All arthritic groups showed higher swelling at all follow-up time points compared with baseline (BA) (*P* < 0.0001). Swelling decreased from FA.1 to FA.2 in all arthritic groups (AIA: *P* < 0.0001; AIA + T: *P* < 0.0001; AIA + E: *P* < 0.0001; AIA + T+E: *P* = 0.0013), followed by stabilization thereafter. Only AIA differed between FA.2 and FA.3 (*P* = 0.0208).

#### Assessment of grip strength (muscle strength)

Grip strength (right hindlimb) differed significantly over time (F(4,130) = 8.84, *P* < 0.0001), between groups (F(4,130) = 3.80, *P* = 0.0129), and for the time **×** group interaction (F(16,130) = 3.02, *P* = 0.0003).

**Fig. 2:**
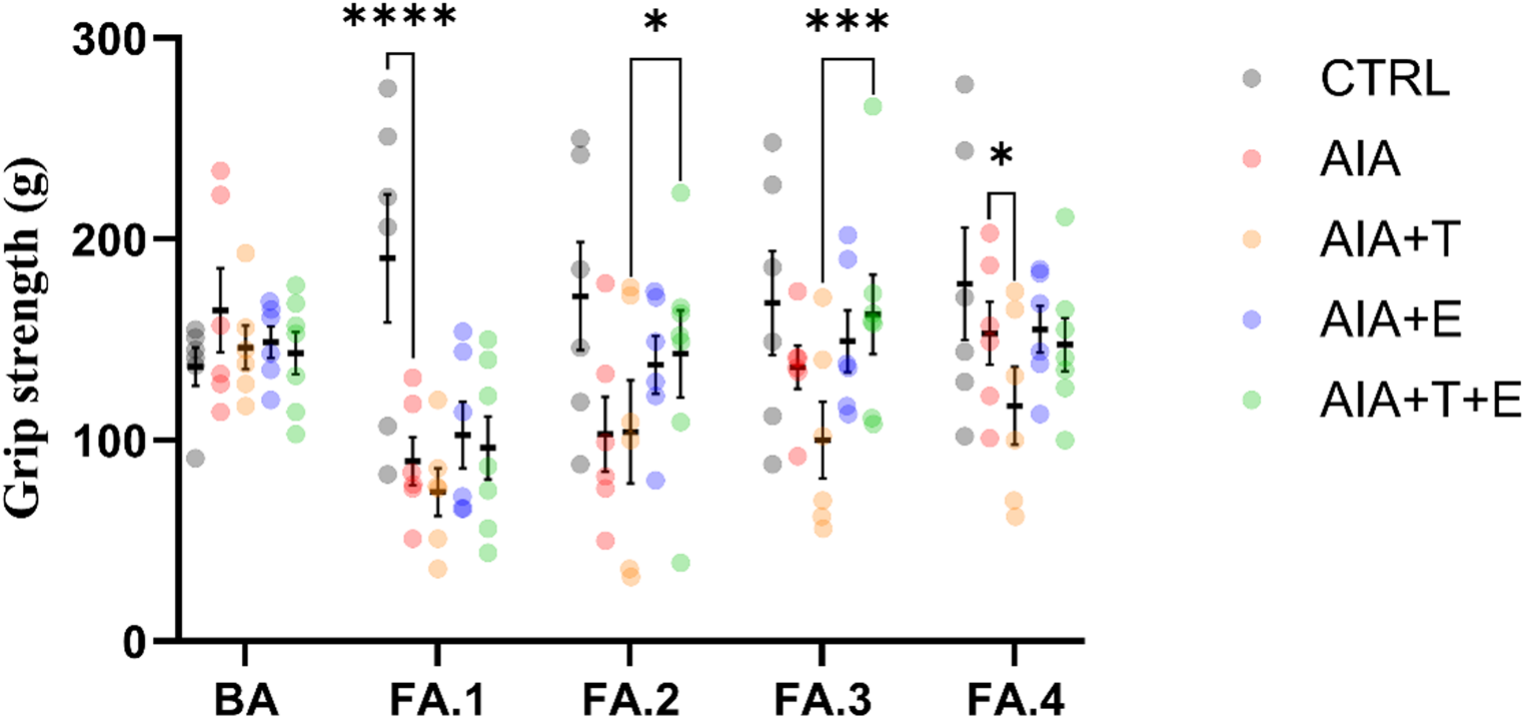
Grip strength (g) in 4-month-old *Wistar* rats assessed in the right hindlimb. Experimental groups: control (CTRL, *n* = 6), adjuvant-induced arthritis (AIA, *n* = 6), AIA + turmeric (AIA + T, *n* = 6), AIA + stair-climbing resistance exercise (AIA + E, *n* = 6), and AIA + turmeric + exercise (AIA + T+E, *n* = 7). Measurements were performed at baseline (BA; Day 1), after induction (FA.1; Day 11), and during follow-up (FA.2–FA.4; Days 15, 19, and 23). Each dot represents one animal (biological replicate). Horizontal bars indicate the mean and vertical bars indicate mean ± SEM. Data were analyzed using two-way repeated-measures ANOVA followed by Fisher’s post hoc test. For clarity, brackets indicate selected key between-group comparisons at the indicated time points (CTRL vs. AIA at FA.1; AIA + T+E vs. AIA + T at FA.2 and FA.3; AIA + T vs. AIA at FA.4); all other comparisons are reported in the Results text. *P < 0.05, **P < 0.01, ***P < 0.001, ****P < 0.0001

AIA exhibited reduced grip strength during the first three follow-up time points (FA.1 and FA.2, *P* < 0.0001; FA.3, *P* = 0.0465), returning to values comparable to CTRL at FA.4. AIA + T showed lower strength than AIA at FA.4 (*P* = 0.0411), whereas AIA + E did not differ from AIA. AIA + T+E showed improved strength compared with AIA + T at FA.2 (*P* = 0.0279) and FA.3 (*P* = 0.0005), but did not differ from AIA + E (Fig. 2).

Within-group analyses indicated no changes in CTRL. All arthritic groups exhibited lower grip strength compared with baseline (*P* < 0.0001). Partial recovery was observed from FA.1 to FA.2 in the exercise-treated groups, including AIA + E (*P* = 0.0480) and AIA + T+E (*P* = 0.0085).

### Morphological and morphometric analysis of muscle fibers

**Fig. 3:**
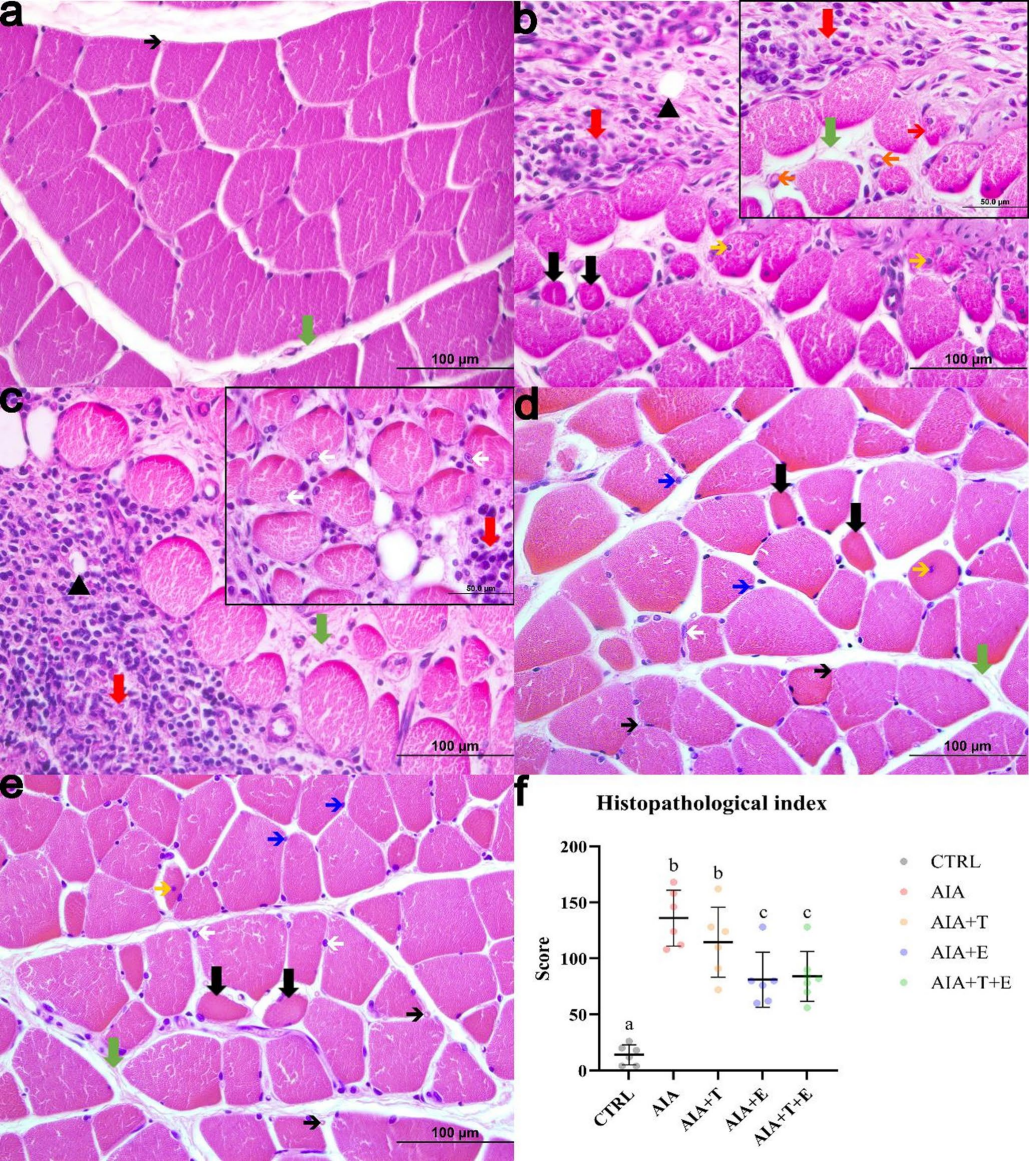
Photomicrographs of transverse sections of the extensor digitorum longus muscle from 4-month-old *Wistar* rats stained with hematoxylin and eosin and examined using a 40× objective. Panels represent the following experimental groups: (**A**) control (CTRL, *n* = 6), (**B**) adjuvant-induced arthritis (AIA, *n* = 6), (**C**) AIA + turmeric (AIA + T, *n* = 6), (**D**) AIA + stair-climbing resistance exercise (AIA + E, *n* = 6), and (**E**) AIA + turmeric + stair-climbing resistance exercise (AIA + T+E, *n* = 7). Green arrow, connective tissue; thin black arrow, capillary. Representative findings include fiber size variability and fibers with centrally located nuclei (yellow arrow) and basophilic halos (white arrow), adipose tissue (black arrowhead), mononuclear inflammatory infiltration/tissue remodeling (red arrow), degenerating fibers (thin red arrow), and congested blood vessels (orange arrow). Insets in panels B and C show higher-magnification views obtained from the same sections. (**F**) Histopathological index. Each dot represents one animal (biological replicate); horizontal bars indicate the mean and vertical bars indicate the standard deviation (SD). Different superscript letters indicate statistically significant differences (*P* < 0.05)

In the control group, extensor digitorum longus (EDL) muscle fibers displayed preserved morphology, characterized by polygonal shape, organized fascicular arrangement, and peripheral multinucleated nuclei (Fig. 3A). In contrast, muscles from the AIA and AIA + T groups exhibited marked morphological alterations, particularly in peripheral regions adjacent to the epimysium. These alterations included mononuclear inflammatory infiltration and areas of tissue remodeling, as well as fiber size variability, fascicular disorganization, and fibers with centrally located nuclei with basophilic halos. Congested blood vessels containing red blood cells and disruption of connective tissue organization were also evident (Fig. 3B–C).

The exercise-treated groups (AIA + E and AIA + T+E) also showed morphological alterations; however, these changes were less pronounced compared with AIA and AIA + T (Fig. 3D–E).

**Fig. 4:**
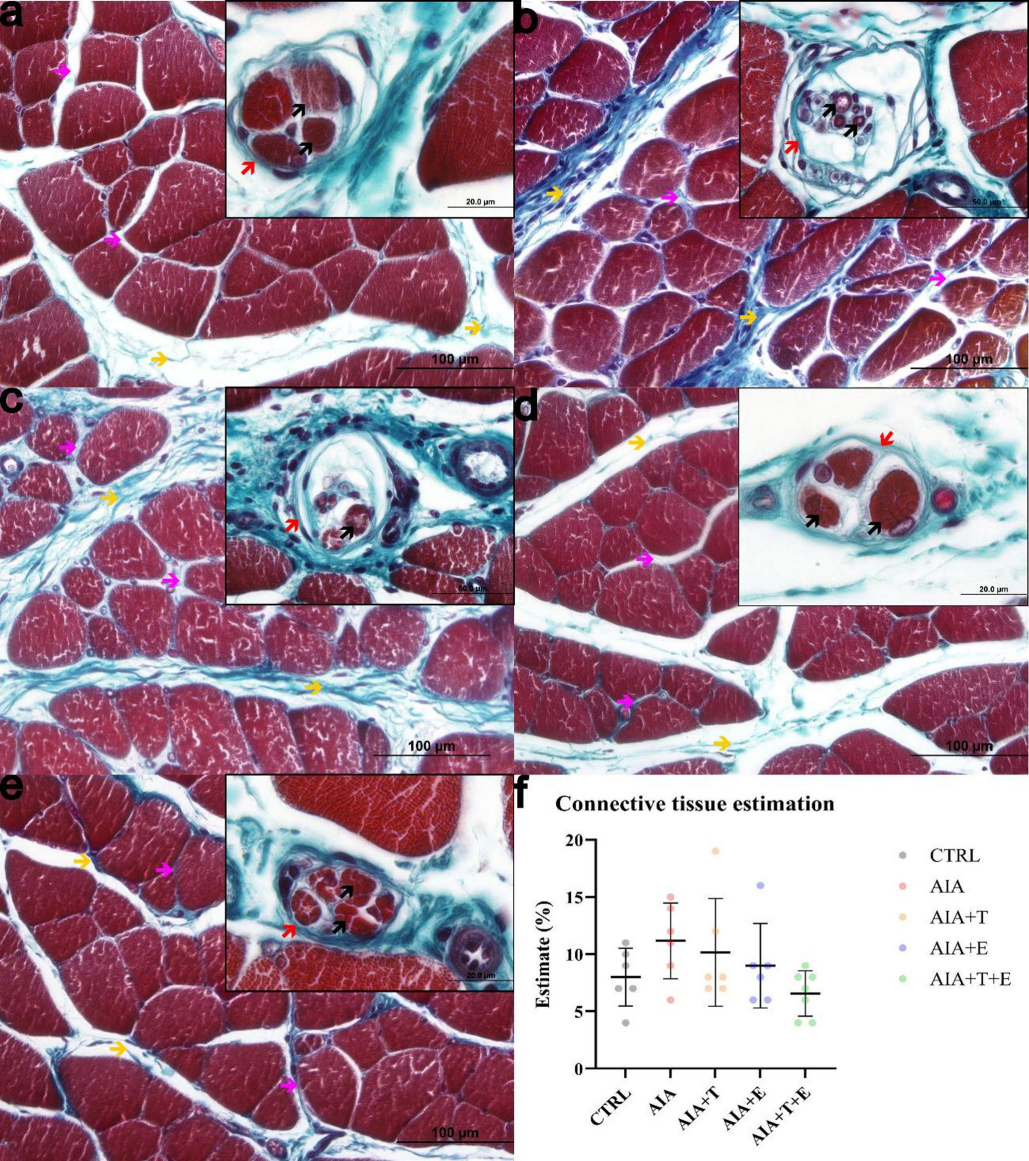
Photomicrographs of transverse sections of the extensor digitorum longus muscle from 4-month-old *Wistar* rats stained with Masson’s trichrome and examined using a 40× objective. Panels represent the following experimental groups: (**A**) control (CTRL, *n* = 6), (**B**) adjuvant-induced arthritis (AIA, *n* = 6), (**C**) AIA + turmeric (AIA + T, *n* = 6), (**D**) AIA + stair-climbing resistance exercise (AIA + E, *n* = 6), and (**E**) AIA + turmeric + stair-climbing resistance exercise (AIA + T+E, *n* = 7). (**A**–**E**) Representative images showing connective tissue in the perimysium (yellow arrow) and endomysium (pink arrow). Insets show neuromuscular spindles composed of a connective tissue capsule (red arrow) and intrafusal fibers (black arrow); all insets were acquired using a 100× objective, and those in panels **A**, **D**, and **E** are shown at higher magnification. (**F**) Quantification of connective tissue. Each dot represents one animal (biological replicate); horizontal bars indicate the mean and vertical bars indicate the standard deviation (SD). No significant differences were detected among groups (*P* ≥ 0.05)

Neuromuscular spindles were observed in all groups, although their integrity varied. In AIA and AIA + T, spindle capsules appeared disorganized, and intrafusal fibers showed degenerative features (Fig. 4B–C). Conversely, the exercise-treated groups preserved spindle morphology, exhibiting more organized capsules and intact intrafusal fibers, resembling the CTRL pattern (Fig. 4D–E).

These findings were supported by the histopathological index, which was significantly higher in AIA compared with CTRL (*P* < 0.0001). AIA + E exhibited lower scores than AIA (*P* = 0.0461), whereas no differences were observed between AIA + T and AIA or between AIA + T+E and AIA + E (Fig. 3F).

Morphometric analysis revealed a 15% reduction in muscle fiber area in AIA compared with CTRL (*P* = 0.0008), along with a 10% reduction in the largest fiber diameter (*P* = 0.0036). In contrast, AIA + E showed a 13% increase in fiber area compared with AIA (*P* = 0.0084). No differences were detected between AIA + T+E and AIA + E; however, AIA + T+E exhibited a 14% increase in fiber area compared with AIA + T (*P* = 0.0047) (Table 2).

Regarding centralized nuclei, AIA showed an 89% increase compared with CTRL (*P* = 0.0002). This parameter was significantly reduced in AIA + T (58% reduction vs. AIA, *P* < 0.0001) and even more markedly reduced in AIA + E (77% reduction vs. AIA, *P* = 0.0001). No differences were observed between AIA + T+E, AIA + T, and AIA + E (Table 2).

Capillarization analysis demonstrated that AIA exhibited a 139% increase in capillary number compared with CTRL (*P* = 0.0025), as well as a 100% increase in the capillary-to-fiber ratio (*P* = 0.0036). No significant differences were observed among the remaining groups (Table 2).

### Connective tissue analysis

Connective tissue was observed in the perimysium and endomysium across all experimental groups (Fig. 4A–E). Quantitative analysis revealed no significant differences in connective tissue content among the groups studied (Fig. 4F).

### Analysis of neuromuscular junctions

**Fig. 5:**
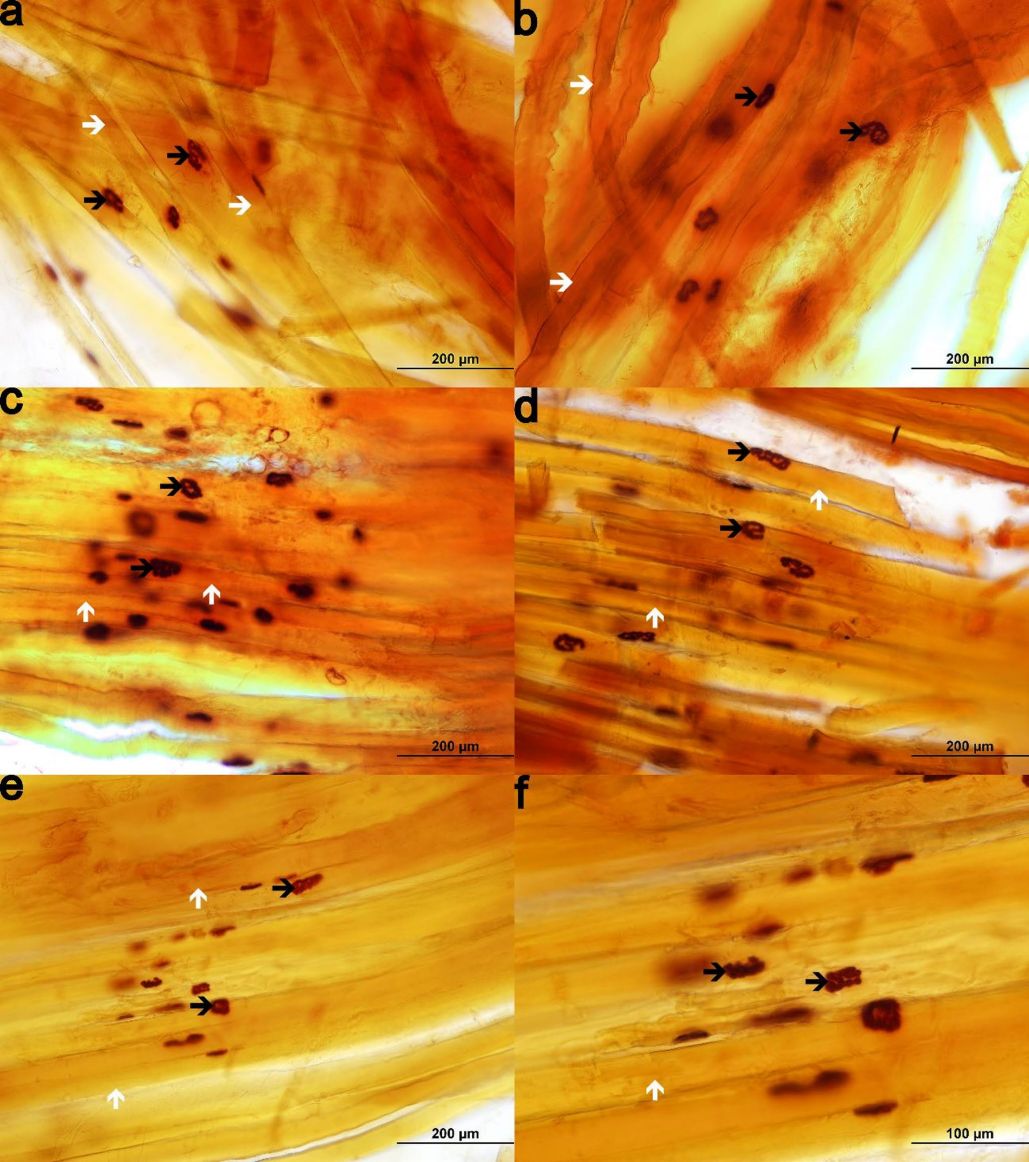
Photomicrographs of neuromuscular junctions from 4-month-old *Wistar* rats. Longitudinal sections of the extensor digitorum longus muscle stained for nonspecific esterase and examined using a 20× objective. Panels represent the following experimental groups: (**A**) control (CTRL, *n* = 6), (**B**) adjuvant-induced arthritis (AIA, *n* = 6), (**C**) AIA + turmeric (AIA + T, *n* = 6), (**D**) AIA + stair-climbing resistance exercise (AIA + E, *n* = 6), and (**E**–**F**) AIA + turmeric + stair-climbing resistance exercise (AIA + T+E, *n* = 7). Representative images show neuromuscular junctions (black arrow) and longitudinal skeletal muscle fibers (white arrow). Oval, elliptical, and round-shaped neuromuscular junctions are observed. Sections were obtained by free-hand cutting, resulting in increased thickness and limited focal depth, which may affect image sharpness but does not compromise morphological assessment. Panel F shows a higher-magnification view obtained using a 40× objective

All experimental groups exhibited oval, elliptical, and round-shaped neuromuscular junctions, with no evident morphological differences between groups (Fig. 5A–F).

Morphometric analysis revealed no significant differences in junctional area or largest diameter among groups. However, AIA showed a 7% increase in the smallest diameter compared with CTRL (*P* = 0.0152), while AIA + E exhibited an 8% reduction in this parameter compared with AIA (*P* = 0.0077) (Table 2).

**Table 2:** Morphometric parameters of the EDL muscle and neuromuscular junctions of 4-month-old *Wistar* rats

Parameters	CTRL (*n* = 6)	AIA (*n* = 6)	AIA + T (*n* = 6)	AIA + E (*n* = 6)	AIA + T+E (*n* = 7)
**Muscle fibers**					
Muscle Fiber area (µm²)^1,3^	1896 ± 106ᵃ	1620 ± 95ᵇ	1530 ± 162ᵇᶜ	1862 ± 173ᵈ	1784 ± 135ᵈ
Larger diameter (µm)^1,3^	66.1 ± 2.9ᵃ	59.6 ± 4.2ᵇ	54.1 ± 5.1ᵇ	58.9 ± 2.8ᵇ	56.8 ± 5.0ᵇ
Smaller diameter (µm)^1,4^	37.9 ± 2.1	36.7 ± 2.6	36.1 ± 3.2	38.7 ± 2.2	37.2 ± 2.2
Number of fibers^1,4^	257 ± 21	289 ± 33	302 ± 33	261 ± 45	297 ± 71
Peripheral nuclei^1,3^	338 ± 72	393 ± 50	360 ± 89	307 ± 64	385 ± 115
Central nuclei^1,4^	3.5 ± 1.4ᵃ	30.2 ± 7.3ᵇ	12.7 ± 6.7ᶜ	7.2 ± 4.4ᶜ	11.1 ± 7.2ᶜ
Nucleus/fiber ratio^1,3^	1.2 ± 0.3	1.4 ± 0.1	1.2 ± 0.3	1.2 ± 0.2	1.3 ± 0.3
Capillaries^1,3^	76 ± 23ᵃ	184 ± 61ᵇ	182 ± 63ᵇ	116 ± 95ᵇ	105 ± 49ᵇ
Capillary/fiber ratio^1,3^	0.3 ± 0.1ᵃ	0.6 ± 0.2ᵇ	0.6 ± 0.2ᵇ	0.4 ± 0.3ᵇ	0.3 ± 0.1ᵇ
Neuromuscular spindles^1,4^	2.7 ± 1.6	2.8 ± 0.8	2.0 ± 1.1	1.7 ± 0.8	2.7 ± 1.1
**Neuromuscular junctions**					
Neuromuscular junction area (µm²)^1,3^	587 ± 75	586 ± 72	548 ± 61	583 ± 71	583 ± 62
Larger diameter (µm)^1,3^	40.1 ± 3.4	40.1 ± 4.1	40.4 ± 2.6	38.5 ± 1.4	40.4 ± 1.5
Smaller diameter (µm)^1,4^	18.2 ± 1.2ᵃ	19.5 ± 0.7ᵇ	18.2 ± 0.7ᵇᵈ	18.1 ± 1.4ᶜ	18.3 ± 1.5ᶜᵈ

CTRL control group; AIA adjuvant-induced arthritis; AIA + T adjuvant-induced arthritis + turmeric; AIA + E adjuvant-induced arthritis + stair-climbing resistance exercise; AIA + T+E adjuvant-induced arthritis + turmeric + stair-climbing resistance exercise; EDL extensor digitorum longus

Values are expressed as mean ± standard deviation. Different superscript letters indicate statistically significant differences (*P* < 0.05)

¹ Student’s t-test (parametric data; AIA vs. CTRL)

² Mann–Whitney test (non-parametric data; AIA vs. CTRL)

³ Two-way ANOVA with Fisher’s post hoc test (parametric data among AIA, AIA + T, AIA + E, and AIA + T+E)

⁴ Kruskal–Wallis test with Dunn’s post hoc test (non-parametric data among AIA, AIA + T, AIA + E, and AIA + T+E)

## Discussion

No changes in body weight were observed in animals with CFA-induced adjuvant-induced arthritis (AIA) receiving the 50 µL dose, consistent with Schnaufer et al. (2023). In contrast, higher CFA doses (100 µL) have been associated with weight loss, possibly due to reduced glucose and leucine absorption (Wang et al. 2017). Therefore, the absence of weight loss in the present study is likely dose-related.

Similarly, no alterations were detected in EDL weight and length. These responses are muscle-specific, and the EDL, due to its predominantly non-postural function, is less susceptible to disuse-related atrophy (Jaspers and Tischler, 1984). Thus, the intrinsic characteristics of this muscle likely explain the lack of gross changes in these parameters.

Functional analyses confirmed marked joint swelling in arthritic animals, a hallmark of inflammatory arthritis, which is largely attributed to elevated levels of pro-inflammatory cytokines such as TNF-α and IL-1β. These cytokines activate NF-κB signaling, leading to increased COX-2 expression and prostaglandin E₂ production (Fattahi and Mirshafiey, 2012). Neither turmeric supplementation (AIA + T) nor stair-climbing resistance exercise alone (AIA + E) was sufficient to reduce joint swelling in the present protocol. The limited effect of turmeric may be related to its low bioavailability when administered as a dry extract (Sasaki et al. 2011). Although stair-climbing resistance exercise has been shown to reduce inflammatory symptoms in other contexts (Gomes et al. 2014), its effects may vary depending on training characteristics such as intensity, volume, and duration (Seo et al. 2014).

Notably, the combined intervention (AIA + T+E) reduced joint swelling compared with AIA + T (and at FA.1 compared with AIA + E), suggesting that combining turmeric supplementation with resistance exercise may enhance inflammatory control. Turmeric has been shown to suppress pro-inflammatory cytokine production (Khan et al. 2022), while resistance exercise promotes the release of anti-inflammatory mediators, which together may contribute to this effect.

Grip strength was reduced in arthritic animals, likely reflecting joint inflammation and pain-related motor impairment (Rannou et al. 2006). Partial recovery observed in the AIA group is consistent with previous findings showing spontaneous functional improvement beyond seven days after CFA induction (de Freitas Tavares et al., 2022). In contrast, grip strength was lower in AIA + T at the final time point, which may relate to effects on excitation–contraction coupling, as inhibition of sarcoplasmic reticulum Ca²⁺-ATPase can impair contraction and relaxation dynamics (Zhang et al. 2021).

Although the exercise-only group did not differ from AIA in intergroup comparisons, progressive intragroup recovery was observed from the second time point onward, which may reflect transient exercise-induced stress followed by adaptive recovery (Smith 2000). In the combined treatment group (AIA + T+E), grip strength was higher than in AIA + T but not different from AIA + E, indicating that stair-climbing resistance exercise was the main contributor to functional improvement. This finding aligns with evidence supporting exercise benefits in rheumatoid arthritis, including improvements in strength, muscle mass, fatigue, and quality of life (McInnes and Schett 2011).

To explore the structural basis of these functional outcomes, morphological analyses were performed. Marked histopathological alterations were observed in EDL muscles of arthritic groups, particularly AIA and AIA + T, including mononuclear inflammatory infiltration/tissue remodeling, fiber size variability, and fibers with centrally located nuclei and basophilic halos. In contrast, the exercise-treated groups (AIA + E and AIA + T+E) exhibited milder alterations, consistent with a protective effect of resistance exercise on muscle structure under inflammatory conditions. Turmeric alone appeared insufficient to induce comparable effects, likely due to the short treatment duration and limited absorption (Sasaki et al. 2011).

Degenerative features and loss of fiber organization were more evident in AIA and AIA + T and less pronounced in the exercise-treated groups, consistent with the histopathological index showing reduced lesion severity with exercise. Loss of fiber conformation may reflect sarcomere remodeling during degeneration (Coutinho et al. 2004), supporting the role of resistance exercise in attenuating inflammation-associated muscle damage.

Congested blood vessels were evident in AIA and AIA + T, which may reflect impaired oxygen delivery associated with hypoxia-related pathways in inflammatory conditions (Ryu et al. 2014). Resistance exercise increases muscle perfusion (Thomas et al. 2020), which may contribute to the comparatively better vascular appearance in exercise-treated groups.

All arthritic groups exhibited nuclei with increased volume and basophilic halos. According to the myonuclear domain theory, each myonucleus governs protein synthesis within a defined cytoplasmic region (Snijders et al. 2021). Enlarged nuclei may therefore represent an adaptive response during muscle remodeling and repair (Crabbs et al.,^2024^).

Morphometric analyses demonstrated reduced fiber area in AIA compared with CTRL, consistent with inflammatory-driven atrophy mediated by cytokines such as IL-6 (Ollewagen et al. 2021). In contrast, AIA + E exhibited greater fiber area compared with AIA, which is consistent with resistance exercise–induced stimulation of protein synthesis and myofibrillar accretion (Lovison et al. 2018).

Central nuclei were more frequent in AIA, indicating ongoing regeneration (Gundersen 2016). The lower number of centrally located nuclei in treated groups (AIA + T, AIA + E, and AIA + T+E) may reflect differences in the stage of muscle remodeling; however, this interpretation should be made cautiously, as the histopathological index reflects overall lesion severity and not centrally nucleated fibers alone.

Increased capillarization observed in AIA compared with CTRL is consistent with TNF-α–mediated angiogenic signaling aimed at facilitating inflammatory cell recruitment (Gravallese et al. 2003). Despite opposing reported effects of exercise (pro-angiogenic) (Gavin et al. 2007) and turmeric (anti-angiogenic) (Bhandarkar and Arbiser, 2007), no additional modulation was observed here, likely due to limited turmeric bioavailability and the short intervention period (Sasaki et al. 2011).

Although no quantitative differences were detected in connective tissue content, inflammatory arthritis can promote structural disorganization in the extracellular matrix, potentially impairing force transmission and regeneration (Sanes 2003).

Few studies have examined neuromuscular spindles in rheumatoid arthritis. In the present study, spindle alterations were more evident in AIA and AIA + T, whereas exercise-treated groups maintained more preserved morphology. Given the role of spindles in proprioception (Zvaritch and MacLennan, 2015), inflammatory cytokine accumulation may contribute to spindle degeneration (Reid and Li 2001). Exercise has been reported to modify spindle structure and reduce inflammatory signaling (Jiménez-Maldonado et al. 2019), which may help explain the comparatively preserved spindle morphology observed in AIA + E and AIA + T+E.

Neuromuscular junction morphology remained largely preserved across groups. However, an increase in the smallest junction diameter in AIA was attenuated by resistance exercise. Oxidative stress in inflammatory and autoimmune conditions can inhibit acetylcholinesterase, leading to acetylcholine accumulation and altered synaptic morphology (Weiner et al. 1994; Araújo et al. 2016). Resistance exercise can reduce oxidative stress and modulate neuromuscular activity (Thirupathi et al. 2021; Jacob et al. 2022), which may contribute to the normalization of junctional dimensions observed in AIA + E.

This study has limitations. Turmeric supplementation alone showed limited effects, possibly due to the short supplementation period and low bioavailability of the formulation used. Previous studies suggest that longer interventions (8–16 weeks) and more bioavailable formulations (e.g., nano-formulations). In addition, spontaneous functional improvement observed in arthritic animals may reflect the acute inflammatory course of the CFA model, which can partially subside over time. Finally, the absence of immune or fiber-type labeling limits more specific interpretation of inflammatory cell populations and regenerative stages. These factors should be addressed in future studies to better delineate the therapeutic potential of turmeric on inflammation-associated muscle alterations.

## Conclusion

Functional assessments indicated that adjuvant-induced arthritis (AIA) negatively affected motor function, characterized by increased joint swelling and reduced hindlimb grip strength. Turmeric supplementation (dry extract) alone showed limited effects on these functional outcomes. In contrast, stair-climbing resistance exercise—either alone or combined with turmeric—attenuated functional impairments, with values approaching baseline levels.

AIA was also associated with structural alterations in the EDL muscle, including inflammatory/tissue remodeling features, degenerative changes in muscle fibers, and alterations in neuromuscular spindles and neuromuscular junction morphometry. Turmeric supplementation alone did not substantially modify these outcomes under the conditions tested. Conversely, stair-climbing resistance exercise, either alone or in combination with turmeric, was associated with a more preserved musculoskeletal phenotype, supporting resistance exercise as a complementary strategy to mitigate inflammation-associated musculoskeletal impairment in this experimental model.

## Data Availability

Data will be made available upon reasonable request.
